# Interpreting chronic disorders of consciousness: medical science and family experience

**DOI:** 10.1111/jep.12220

**Published:** 2014-07-04

**Authors:** Andrew Edgar, Celia Kitzinger, Jenny Kitzinger

**Affiliations:** 1 Centre for Applied Ethics, Cardiff UniversityCardiff, UK; 2 Department of Sociology, Wentworth College, University of YorkHeslington, UK; 3 JOMEC, Cardiff UniversityCardiff, UK

**Keywords:** CDoC, communication, interpretative competences, patienthood, philosophy, vegetative states

## Abstract

**Rationale, aims and objectives:**

Chronic disorders of consciousness (CDoC) pose significant problems of understanding for both medical professionals and the relatives and friends of the patient. This paper explores the tensions between the different interpretative resources that are drawn upon by lay people and professionals in their response to CDoC.

**Methods:**

A philosophical analysis of data from 51 interviews with people who have relatives who are (or have been) in a vegetative or minimally conscious state.

**Results:**

The medical specialist and the lay person tend to draw on two different interpretative frameworks: a medical science framework, which tends to construct the patient in terms of measurable physical parameters, and an interpretative framework that encompasses the uniqueness of the patient and the relative's relationship to them as a social being.

**Conclusions:**

These differences potentially lead to ruptures in communication between medical professionals and relatives such that that an increased self-consciousness of the framing assumptions being made will facilitate communication and enrich understanding of CDoCs.

## Introduction

Chronic disorders of consciousness, such as coma, persistent/permanent vegetative states (PVS) [1] or unresponsive wakefulness syndrome [2], and the minimally conscious state (MCS) [3], pose profound problems of interpretation for lay people and medical professionals alike. For the lay person, such as the relative or friend of the patient, a chronic disorder of consciousness (CDoC) seems to defy many of the categorical distinctions through which sense is routinely made of everyday experience. A patient in a vegetative state seems to be neither unambiguously alive nor dead [4], and one in a MCS may seem neither conscious nor unconscious, neither an agent nor a passive object. The very continuity between who the patient is now and who they were prior to the onset of the condition can be profoundly enigmatic. Fundamental to this is the problem of how one can gain an insight into the state of consciousness of the patient merely from observation of their physical movements – the flicker of an eyelid, gaze, thrashing or kicking. The diagnosis and prognosis of CDoC remains complex and problematic, despite the introduction of technically advanced diagnostic tools such as brain imaging techniques [5]. Given the confusion potentially experienced by relatives and a degree of uncertainty inherent in medical science, diagnosis and prognosis can be sites of significant conflict and misunderstanding.

This paper will explore the tensions between the different interpretative resources that are drawn upon by lay people and professionals in their response to CDoC. It will be suggested that the lay person and the medical specialist, while confronted by a common problem of interpreting the inherently equivocal physical movements of the patient, tend to draw on two different interpretative frameworks that shape (and are shaped by) fundamentally different approaches to the problem. It will be suggested that the medical science, upon which the professional draws, tends to construct the patient in terms of measurable physical parameters that have statistically predictable consequences. In contrast, the relative requires an interpretative framework that will encompass the uniqueness of the patient and their relationship to them, and thus a framework that constitutes the patient as a continuing member of a community – as a social being. While it cannot be argued that the interpretations and judgements of medical professionals are wholly determined by the scientific frameworks – indeed it must be acknowledged that many strive to adopt the family's perspective – it will be suggested that there is a tension between a scientific or biomedical model of CDoC and a social and relational experience. The argument is not that one or the other of these frameworks is false. Rather, that they are complementary. Nonetheless, a failure to recognize this interpretative tension and complementarity may serve to inhibit both dialogue between relative and physician, and the medical professional's own articulation of the patient's condition, and this in turn inhibits an understanding of what the goals of medical care should be.

The paper draws upon a database of interviews with the relatives of patients who are (or have been) in a vegetative state (VS) and a MCS. This research has been carried out by sociologists at the Universities of York and Cardiff, led by Celia Kitzinger and Jenny Kitzinger, whose own sister, Polly, was severely brain injured and was in a VS and then MCS for some time, although she subsequently emerged into full consciousness, with profound and multiple mental and physical disabilities [6]. Beginning in November 2010, 51 participants, all of whom currently or in the recent past had relatives in VS or MCS, were interviewed, typically on a one-to-one basis. The majority of interviews lasted between 2 and 4 hours (with breaks). Uniform structures were not imposed upon the interviews, thereby allowing participants to tell their stories in their own ways. The transcribed, and rigorously anonymized, interviews were analysed in order to identify thematic patterns [7].

## Defining PVS

The term ‘persistent vegetative state’ was originally proposed by Jennett and Plum to label a state of ‘wakefulness without awareness’ [8]. It is ‘a complex neurological condition in which patients appear to be awake but show no sign of awareness of themselves or their environment’ [1], or as one interviewee (a brother of a patient in PVS) puts it: ‘basically your body is working on autopilot, it breathes, it farts, it feeds but it consciously can't do any of it’. A coma patient will not manifest the sleeping–waking cycle of the patient in VS, while the patient in MCS will manifest fluctuating degrees of awareness of their self and their environment, for example by responding to stimuli [9].

It is the ambiguity of CDoCs, in which states that are usually conjoined – wakefulness and awareness – are in fact separated, that disrupts the possibility of both coherent lay and medical understanding of the condition and its consequences. As a mother of a patient in PVS expresses this:

I never in my wildest imagination – I'd never seen anything like this, you know. And I don't think most people have seen anything like that. It's not something that you'd, you know, come across beforehand. Disability, yeah. But as far as I'm concerned, this isn't a disability. It's a devastation.

This suggests that the incomprehensibility of the situation adds a particular dimension to the relatives’ distress. The cultural resources through which one routinely makes sense of experiences, and through which one therefore knows how to go on, practically, living a coherent social life, are found wanting. It is not merely that there are no obvious categories by which to organize one's experience, but also no socially recognizable rites or practices (akin to baptisms and funerals) through which one may negotiate the transition to this new state. This failure lies, in part, in the categories through which meaningful experience is constituted, such as life and death or consciousness and unconsciousness.

## Understanding the patient

Medical diagnosis may not help to relieve the senselessness experienced by the relative. That is to say that diagnosis may not offer a suitable categorical framework within which the condition and its consequences may be understood. While the various disorders of consciousness may be defined readily enough, diagnosis itself remains highly problematic, with surveys having suggested that some 40% of patients may have been misdiagnosed as being in PVS when actually in MCS [10].

Inaccurate diagnosis may be attributed to a number of factors, including the low incidence of the condition, and thus the physicians’ unfamiliarity with it, the lack of systematic training in the use of diagnostic tools and even variation in the tools themselves [1]. Newer diagnostic tools, such as the Coma Recovery Scale – Revised (CRS-R) [11] tend to be more sensitive than the older Glasgow Coma Scale (GCS) to differences between PVS and MCS, thus helping to improve diagnosis. The root problem may nonetheless be understood in terms of the gap that the conditions open up between physical movement and intentionality. PVS turns the abstract philosophical problem of other minds – which is to say, how do we know that the physical bodies that we encounter around us bear minds or consciousnesses like our own – into a pressing practical problem. As competent social agents, we have rich, if tacitly understood, skills in interpreting the behaviour of others, and in distinguishing intentional from unintentional gestures. Consider seeing someone wink, and consider the sort of considerations one uses in order to understand that it is an intentional and meaningful gesture – such as an expression of collusion and complicity – as opposed to being a mere tick. One will draw upon knowledge of the personality of the person winking, and on social and physical contexts in which the behaviour occurs, recognizing, for example, that you and the one who winks have something to be in collusion about, you are not strangers to each other, and that it is the sort of situation in which a wink is appropriate because, say, you are required to be silent or you are protecting a playful conspiracy from a third party [12]. Crucially, human actions, be they mere physical behaviours or complex verbal expressions, are not inherently meaningful. They make sense because they allow an interpreter to recognize or construct often subtle references to context. They are indexical.

Patients in VS and MCS do not offer movements that yield themselves to ready interpretation. Relatives are well aware of the problems and dangers of interpreting their relative's movements. Thus, the wife of a patient comments that: ‘He'll grip things, but it's very difficult to know if these are conscious decisions or if he's just you know, like a baby, his grasp reflex, it's hard to know’, or again a mother notes that, as her son came out of a medically induced coma, ‘we started to do what all relatives do in these circumstances, you look at every involuntary twitch, every sigh, every flutter of the eyelids and you think that means something’.

The problem may be understood, firstly, in that the sleeping–waking cycle, that for most people is indicative of a complex awareness of one's environment, cannot be so interpreted for the patient in PVS – that is precisely the nature of the condition, with wakefulness being sundered from awareness. Further, the context within which the patient acts, and to which they might be intentionally reacting, is typically impoverished compared with that encountered in everyday interaction. The interpreter, be it the physician or the relative, may perceive the context simply in terms of isolated and quite simple stimuli – the sound of a voice, the touch of a hand and pressure of fingers. Further, the affect that the condition has upon the patient's personality, and thus the continuation of personality traits, is highly uncertain – not least because the recognition of continuity in personality depends precisely upon the capacity to make clearly indexical expressions that has been undermined by the condition. The possibility of meaningful expression is thus distorted and curtailed. Spasticity, along with medical treatments such as a tracheotomy, or indeed physical impairments such as deafness, blindness and paralysis, may significantly inhibit the patient's ability to express themselves. Finally, but importantly, awareness and the expression of awareness in MCS is fluctuating, making the diagnostic distinction between VS and MCS difficult and contestable. Patients in VS may have a single, isolated moment of lucidity among months or years of apparent unresponsiveness.

Two types of instruments have been developed to tackle this problem: behavioural tools and brain imaging. Behavioural tools, such as the GCS, the Sensory Modality Assessment Technique (SMART), the CRS-R and the Wessex Head Injury Matrix, typically serve to systematize the observation of the patient's responses and movements, and the interpretations that can be made of them (see [3] for an overview of these tools). As such, they may be understood as a systematic reconstruction of the sort of observational techniques that the lay person, and indeed the expert physician, might use in trying to elicit evidence of awareness. CRS-R, for example, requires repeated observation of the patient, with their behaviour recorded on six dimensions: auditory, visual, motor, oromotor/verbal functions, communication and arousal, with each dimension scored in terms of the presence or absence of responses to specific sensory stimuli (Brainstem reflex is also recorded, but not scored) [3],[11]. CRS-R is specifically designed to distinguish between patients in PVS and in MCS. SMART similarly requires observation of responses to standardized sensory stimulation, focusing on vision, hearing, taste, touch and smell, and seeks to bypass problems created by such physical incapacities as the blindness or deafness of the patient, and significantly involves relatives, friends and lay and professional carers in the assessment process [13].

No tool can directly access the patient's consciousness or quantify the degree of awareness. Tools, rather, are designed to close the gap between the observable behaviour of the patient and their inner mental state, specifically by simplifying the context within which gestures are interpreted, and thus ascertaining whether physical movements are indeed indexical gestures. Evidence of awareness and intentionality is detected by re-establishing the link between movements and contexts. However, contexts are now primarily the simple, isolated and readily observable stimuli provided by the tester, not the complex and often ambiguous contexts with which competent social agents engage. The physical movement of the patient can then be interpreted, with a minimal degree of ambiguity or contestability, as a response to a given stimulus (or as a failure to respond). The patient becomes, as it were, a black box, assessed in terms of a quasi-causal link between stimulus and response, and not as a competent social actor.

Observational tools work by recreating a highly stylized version of the routine social competences through which we interpret behaviour as meaningful. This may be justified, in part, on the assumption that the patients themselves, because of their condition, lack the competence any longer to engage with complex contexts. Thus, such tools refine and systematize observation, stripping away, as it were, some of the noise that inhibits interpretation.

It might be suggested that brain imaging, through such techniques as electroencephalography (EEG), positron emission tomography or functional magnetic resonance imaging, offers an opening of sorts into the black box. While debates remain about the degree to which such imag(in)ing serves to improve diagnosis and prognosis (see [1] ), it is perhaps most significant in seemingly allowing the diagnostician to bypass the patient's physical limitations. While the physical behaviour of the patient may be compromised, inhibiting communication and expression, imaging turns to the even more alien behaviour of the brain – as represented by the various imaging techniques – and again seeks a way of ascribing meaning to it. Research by Monti *et al*. sought to test whether certain patients diagnosed as being in PVS were responding to verbal commands, such as to imagine that one is playing tennis, by identifying similarities between imaged brain activity in the patients and in healthy control subjects [9]. Demonstrating that patients diagnosed as being in PVS may nonetheless have awareness, albeit not physically expressed, indicates that the absence of (behavioural) evidence of awareness does not necessarily entail evidence of absence of awareness.

Some relatives of patients in VS and MCS express a certain ambivalence towards behavioural tools and brain imagining techniques [14]. The results of behavioural observations and imaging techniques are questioned, but along a number of different parameters. In some cases, there is a simple scepticism about how good the scientific knowledge of the brain is, noting, for example, the brain's complexity or hidden nature in comparison with more routine and overtly observable medical problems such as a broken arm. It may be argued that, more fundamentally, the biomedical framework, within which most diagnostic tools are framed, may make their results appear alien to the relatives’ experience, and indeed of only limited relevance in their struggle to make sense of the situation, and to meaningfully go on in their social practices.

At one pole, the supposed rigour of a scientific method appears, to some family members, simply not to work, or to let them down. Thus, it has been suggested earlier that the point of behavioural tools is to systematize observational recording, yet relatives are aware of inconsistencies between different observers. As one father notes, ‘I do accept that it does come down to interpretation, someone's response or lack of response to a stimulus. It's not the same as finding out why an engine doesn't work’. This effectively summarizes the interviewee's earlier comment:

I've never seen the details of how a SMART test is supposed to be performed and interpreted. But this – if you're going to apply a scientific method to assessing someone's condition, the whole point of it is that it's consistent and repeatable and independent of the person who's carrying out the tests. So why are we getting different answers from different people?

In contrast, some relatives may find the supposed objectivity of tests problematic. They are thus wary of test results, suggesting that such tests can be insensitive to the particular condition and circumstances of the patient. Relatives suggest that a recent movement between hospitals will have tired the patient and so made them unresponsive; or they may currently be undergoing a drug treatment that will suppress awareness or hamper its expression. Others, although accepting the objectivity of tests, nonetheless insisted that they cannot measure everything.

The objectivity and scientific status of tests was also seen as something of a double-edged sword. Relatives note the danger of being on the ‘wrong side’ of a test, which is to say that the test gives results that frustrate one's hopes and expectation. As one relative notes:

Do you know, like I thought all these things [i.e. EEG] were just benefits, and if you didn't get the result you were looking for then, so what? It doesn't mean anything, do it again later. But I didn't realize how it could actually work against you.

The presupposed objectivity, and thus superiority, of scientific evidence may be resented as trumping the observations, and even intuitions, of the relatives. In the case of the relative just quoted, she goes to significant lengths to document her own observations of the patient, including recording videos of him, in an attempt to resist the official medical interpretation that she fears is being imposed upon him.

It may then be suggested that the supposed objectivity of the scientific tests makes their results appear alien in the eyes of some relatives and to the subjectivity and humanity of the patients. While, as noted earlier, SMART tests do take into account the relatives’ perspectives, and may be welcomed as such by relatives, in some cases, the relatives’ responses to even SMART test results may be understood as expressive of the relatives’ perception of the results as being too coldly objective.

While tests might more or less successfully label patients, they may do little, if anything, to help make sense of the particular experience, and crucially, the ‘devastation’ felt by the relatives. While ‘PVS’ or ‘MCS’ might have meaning as medical categories – and indeed will have significant consequences for the therapies to be prescribed (or not), and will have legal implications – they do little to touch upon the complex social problem that is the CDoC.

## Frameworks of interpretation

It may be suggested that, as frameworks through which the patient's condition are to be understood and constructed, diagnostic tools typically fail to engage sufficiently with the experience of relatives. This is, crucially, not to criticize medical professionals who use such tools, who may and frequently do strive to achieve that engagement. It is rather a recognition of the necessarily one-sided and specialist interpretation that such tools make of the patient's condition. It may be suggested that, on the one hand, medical categories fail to recognize the continuing significance of the personality of the patient, and on the other hand, they fail to recognize that the patient continues to be a social being, bound up in the social relationships of relatives and friends.

The potential that tension may result between relatives and physicians is frequently expressed vividly in the relatives’ stories. A wife protests against the apparent reduction of her husband to ‘a lump of meat’: ‘this is a person we're talking about, not just a thing. It's not a lump of meat, you know, it's a person who's got a future and has grandchildren and children and a wife that needs them, you know.’ Another women recalls emphatically defending the patient as ‘a man, he's my partner, I've got a baby, he's my baby's dad’, when the consultant in the intensive care unit reportedly insisted that he ‘will not keep a slab of meat, that's all he will be, a slab of meat, alive in my room’. The continuation of the patient as a person, with a future and with social ties, is thus asserted, in part, against the threat of any form of medical reductionism (and in part because of a hope against hope of recovery – as in the insistence on both the present tense ‘he's my baby's dad’ and the assertion that the patient is someone ‘who's got a future’).

It should, of course, be pointed out that this consultant's behaviour (as reported by the interviewee) was extreme, and by no means all consultants were reported to show such insensitivity. Yet the consultant's point (if not the way it is expressed) may, in medical and ethical terms, be defensible. If the patient has a poor prognosis, then the attribution of personhood to them may seem to be fanciful, and indeed an illusion of which the relatives need to be disabused in the best interests of the patient. Relatives react differently, and while some suggest that they would welcome such honesty, others require the medical staff to continue to acknowledge the patient's (current and future) personhood. It may be suggested that the continuing personhood remains important to some relatives precisely as a way of coping with and moving on with the crisis. It is, indeed, not an illusion, but a social reality sustained through their interaction with the patient. If the patient and their predicament can be understood through the category of ‘person’, with an attendant hope of recovery, then the situation retains a vestige of everyday meaning, and the relatives can continue to act towards the patient – and interact with each other – purposefully. Strict medical diagnoses and prognoses may strip away this meaningfulness, leaving an incomprehensible void, or leaving family members believing that the only reasonable response would be to allow death, when often (once the patient has stabilized) the only way of guaranteeing death is the withdrawal of artificial nutrition and hydration – an option considered totally intolerable by many families [15].

The continuation of meaningful interaction, grounded in a sense of continuing personhood (or at least allowing for that possibility), may be seen when relatives strive to maintain the social life of the patient. Relatives talk to the patient, as if the patient was aware and a participant in a conversation. Further, relatives tend to the patient's appearance. For example, a patient's facial hair is plucked by relatives ‘because she would have been horrified to sit there with hairs on her face … and she did have a lot of friends still coming to see her’. Others ensure that the patient is not left in bed all day, ensuring a form of socializing that goes beyond avoiding medical problems such as lung infections, taking them to sit in a common room or to ‘join’ a social event. Others take their relatives out: ‘going outside and trying to present her, when we were outside trying to dress her and present her in a way that was consistent with her – with how she would have done anyway’. The appearance or presentation of the patient, as the most basic element of their interaction with others, and thus of their social being, remains important as a focus for meaningful action and the sustaining of their personhood.

The differences in ascription and construction of the patient's personality will have consequences for the way in which the relatives evaluate the condition and treatment of the patient. The medical framework tends to focus on predicting and improving the neurophysiological functioning of the patient. To gain functionality is an improvement. Yet relatives may reject this reasoning, questioning the ‘clearly defined hierarchy’ that doctors take for granted. While a full recovery of consciousness may be desirable in many cases, the transition from VS to MCS can be perceived as placing an additional burden on the patient, precisely as they acquire some awareness of their condition, as one interviewee expressed it: ‘this vital, intelligent, sensitive man trapped in his body and mind. Unable to communicate or move and visibly distressed by this’.

More precisely, the formulation of what the patient ‘would have wanted’ and/or appears currently to want will determine for the relatives, very specifically, what is good or bad treatment for this particular person. The healthy self, with full comprehension, is imagined, and sometimes witnessed, to be experiencing and judging the self as patient, who has no or very little awareness. What counts as ‘a good prognosis’ is not then determined in the abstract – in terms of a medically defined improvement in functioning. Rather, the particular patient's apparent horror at the undignified condition that they are forced to endure, or conversely, their apparent bravery and determination to recover, frames the relative's perception of what is a desirable outcome for this *particular* patient. Whether the ascription of these wants and judgements to the patient is an accurate assessment (for relatives may be projecting their own hopes and frustrations, as much as reconstructing the patient's pre-illness personality or current wishes) may not be to the point.

## Conclusion

It has been argued earlier that CDoCs typically pose a fundamental challenge to lay people and medical professionals alike. Physical movements are often the only clues – bare and often ambiguous as they are – to the potential inner mental life of the patient, if any exists. The interpretation of these movements as being expressive or otherwise of the patient's meaningful intentions has significant consequences, not merely for the medical treatment of the patient, but also potentially for their legal and social status. Earlier it was suggested that behavioural diagnostic tools systematically refine and simplify the observational competences that social agents use in everyday life in order to recognize intentional behaviour, thereby striving to make judgements based upon such competences valid and reliable. If successful, they perform a vital role in understanding the condition of the patient. Yet it was suggested that, precisely in systematizing a lay social competence, such tools were in danger of isolating the patient from richer social relationships and abstracting away their particular personalities (SMART tests, when used well, may be seen to be exemplary in striving to avoid this, by including the perspectives of the patient's relatives, friends and carers). It was suggested that relatives may, under some circumstances, nonetheless find the results of behavioural and also brain imaging diagnostic tools problematic. It is suggested that the reason for this tension lies in the different interpretative needs of relatives and the medical profession, and thus in drawing upon different interpretative frameworks.

Many relatives try to make sense of the crisis that has befallen the patient by finding ways to include them in meaningful social practices. In such circumstances, the patient is constituted, through the actions of relatives and friends, as a continuing person. It is precisely the particularity of this person, who is still situated and sustained within meaningful social relationships, that diagnostic tools may lose sight of. In their very systematization of lay social competences, they may abstract away something of the complex social context and personality within which the patient's actions and potential aspirations and needs have to be interpreted. Medical professionals will, typically, be sensitive to this problem, and strive to compensate. It may nonetheless be suggested that it is important for the difference between interpretative frameworks to be explicitly acknowledged, and their complementary roles better understood. Only thus will the hindering of the relatives’ capacity for making sense of their situation be prevented. Positively, this also allows medical professionals, lawyers and the relatives themselves to come to an appropriately robust understanding of the patient's social, medical, legal and ethical status.
